# Clinical Anatomy and Diagnostic Challenges in Peripheral Nerve Trauma for the Forensic Physician

**DOI:** 10.3390/diagnostics15131597

**Published:** 2025-06-24

**Authors:** Sorin Hostiuc, Oana-Mihaela Ciobanu, Eliza Popa, Raluca Căținaș, Amalia Maria Ionescu-Mihăiță, Andreea Sima, Ionut Negoi, Mihnea Costescu

**Affiliations:** 1 Department of Legal Medicine and Bioethics, Carol Davila University of Medicine and Pharmacy, 020021 Bucharest, Romania; oana-mihaela.ion@drd.umfcd.ro (O.-M.C.); rmcatinas@gmail.com (R.C.); 2 National Institute of Legal Medicine, 042122 Bucharest, Romania; eliza.popa@inml-mm.ro (E.P.); amalia.ionita@inml-mm.ro (A.M.I.-M.); andreea.sima@inml-mm.ro (A.S.); mihneacostescu@yahoo.com (M.C.); 3 Department of Surgery, Carol Davila University of Medicine and Pharmacy, 020021 Bucharest, Romania; negoiionut@gmail.com; 4 Department of Pharmacology, Carol Davila University of Medicine and Pharmacy, 020021 Bucharest, Romania

**Keywords:** peripheral nerve trauma, legal medicine, forensic pathology, malpractice

## Abstract

Peripheral nerve injuries represent a significant challenge in legal medicine, and their proper management and evaluation are at the intersection of clinical medicine, anatomical science, and legal medicine. In this review, we aimed to integrate current knowledge about the anatomy, physiology, clinical management, and paraclinical assessment of peripheral nerve injuries, targeted explicitly for medical–legal practice. We conducted a comprehensive review of the medical–legal evaluation framework needed to evaluate peripheral nerve injuries, with particular emphasis on anatomical variations, imaging techniques, and methods to assess the timing of injury. Peripheral nerve injuries should be analyzed using a complex approach, which includes anatomical characteristics, variants, microanatomy, physiopathology, imaging, and other paraclinical evaluations. The analysis of causation and timing of injury should be heavily based on objective criteria and should be performed using a reproducible, objective, and scientifically based approach.

## 1. Introduction

Peripheral nerve injuries (PNIs) are often found in patients requiring medical–legal evaluation, and their proper management and expert analysis are usually lacking [1]. Their analysis sits at the intersection of clinical medicine, anatomical science, and legal medicine, often requiring a multidisciplinary approach for proper management.

PNIs are among the most prevalent, yet still understudied consequences of trauma, mainly affecting younger male patients. Aman et al., in an epidemiological analysis of more than 110,000 subjects from a European level 2 trauma center, found PNIs in around 5000 cases (4.5%), with 52.2% being acute PNIs, and the rest non-traumatic or posttraumatic—compression, neuroma formation, tumor, irritation, inflammation. In 46.6% of all cases, they found intact continuity of nerves despite perioperative loss of function; in 7.4%, they found an incomplete laceration, and in 44.8%, a complete nerve laceration. Most injuries were located in the upper extremity (88.3%), affected males (73.5%), and had a mean age of 43.2 [2]. In a larger cohort of 1,230,362 patients, Padovano et al. found a similar incidence of PNIs (2.6%), which included only traumatic cases [3]. Other studies have reported much lower values; for example, a registry-based research from Sweden found an overall incidence of 15.6 per 100,000 cases in men and 10.1 per 100,000 cases in women, with the most affected part being the hand [4]. PNIs are often associated with long-term consequences, extending far beyond acute disability, especially in younger groups [5], causing long-term disability, an increased number of sick days, and even permanent disability pension [6]. They also impose a significant socioeconomic burden, with the average direct medical cost exceeding EUR 4800 per case in Germany (with much higher values for cases with combined tendon or vascular injuries), not accounting for lost productivity and rehabilitation-related expenses [6].

The incidence of traumatic PNIs varies by location and depends on the type of traumatic event. For example, a study by Huckhagel et al. centered on lower limb TPNIs and found the highest overall incidence in motorcycle accidents, followed by car occupants, high falls, and pedestrians. In motorcycle accidents and among pedestrians, the most affected nerve was the peroneal nerve (35.5% and 12.7%, respectively). In car occupants, the tibial nerve was the most affected (32%), and in high falls, low falls (<3 m), and bicycle accidents, the femoral nerve was most affected (13.5%, 5.8%, and 4.8%, respectively) [7].

The clinical management and forensic evaluation of PNIs are based on anatomical principles. Peripheral nerves exhibit significant microarchitectural complexity, with a highly complex fascicular organization, layers of connective tissue, and vascular networks affecting injury patterns and regenerative potential [8,9]. For example, the tensile strength of the epineurium determines susceptibility to traction injuries, whereas fascicular topography has essential consequences for functional outcomes after partial nerve transection [10,11]. Anatomical variations represent a particular diagnostic and medical–legal challenge, especially as they are sometimes related to malpractice claims. About 15–20% of individuals have non-textbook nerve branching patterns or anomalous courses, potentially mimicking pathological findings, complicating surgical exploration, or causing an improper diagnosis.

The medicolegal evaluation of PNIs requires proper integration of anatomical knowledge, clinical expertise, and legal and forensic standards. Two main scenarios dominate medical–legal practice: the assessment of alleged medical malpractice in managing nerve injury and determining injury causation and severity in personal injury claims [8,12]. This review aims to integrate current knowledge about the anatomy, physiology, clinical management, and paraclinical assessment of PNIs for medical–legal practice.

## 2. Fundamental Anatomical Principles of the Peripheral Nerves: Clinical and Medical–Legal Consequences

**Figure 1:**
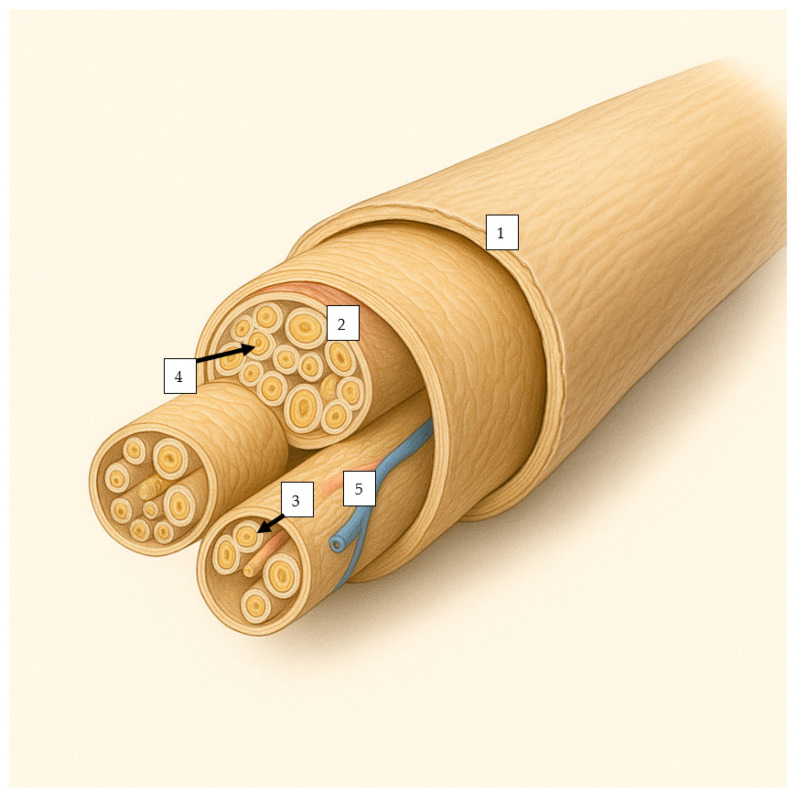
The overall structure of a nerve, containing (1)—an outer layer (epineurium), encasing the nerve, (2)—an intermediary layer (perineurium), encasing multiple nerve fibers, and (3)—an inner layer (endoneurium), encasing each nerve fiber (4). Within the nerve fiber are present nutritional vessels (5).

Peripheral nerves are highly complex anatomical structures that transmit chemical messages between the central nervous system and peripheral effectors. Each nerve consists of three concentric connective tissue sheaths: epineurium, perineurium, and endoneurium, which provide structural integrity and controlled flexibility [13,14]. The epineurium (Henle’s sheet) encases each nerve fiber, creating the most intimate protective sheath for axons and their associated Schwann cells [15]. Schwann cells (mainly) secrete a continuous basal lamina, which has various roles, including Schwann cell proliferation, migration, myelination, and formation of Ranvier nodes, and provides mechanical support [16]. Individual fibers are then bundled together in fascicles, each surrounded by a stronger protective layer, the perineurium, which consists of 7–8 concentric layers of connective tissue, whose cellular component is mainly represented by myoepithelial cells [17]. The perineurium is a selective barrier, controlling molecular exchange between neural tissue and the surrounding environment [17,18]. Multiple fascicles are then bundled together with the associated blood supply and fatty tissue by the epineurium. Peripheral nerves usually contain 3–12 fascicles, depending on the anatomical location, with larger fascicle diameters found in motor nerves compared to sensory nerves (600–800 µm compared to 400–400 µm) [19]. The epineurium consists mainly of longitudinal type I and III collagen fibers, with a few elastic fibers to increase elastic support [20,21]. The epineurium consists of 30–75% of the nerves’ cross-sectional area [18,22], and it houses the *vasa nervorum*, a vascular network consisting mainly of arterioles with 50–150 µm [22]. See Figure 1.

This microanatomical organization of peripheral nerves ensures significant biomechanical resilience, enabling normal function during daily activities and protection against mechanical injuries [15]. The Schwann cell basal lamina ensures the biomechanical protection of individual fibers, which strengthens it in conjunction with specialized proteins such as PMP22 [15]. The perineurial layer further increases mechanical strength and protects from chemical byproducts associated with trauma [17]. Finally, the epineurium offers significant strength and security, accommodating the mechanical limb movement and positioning stresses [23].

Peripheral nerves have a dual vascular supply: extrinsic, represented by segmental arteries from adjacent vascular bundles (e.g., radial collateral artery for the radial nerve), and intrinsic, represented by longitudinal anastomoses within the epineurium. This redundant system allows the nerves to tolerate significant vascular compromise (up to 50%) before ischemic damage occurs [24,25,26]. However, there are areas where this vascular redundancy is minimized, such as the mid-humerus level for the radial nerve or the fibular neck segment for the common peroneal nerve [27,28,29].

Approximately 25% of individuals have significant peripheral nerve variations that deviate from classical anatomical descriptions. If they are not known, there is an increased risk of damage during the surgical procedure or improper management of the symptomatology associated with the injury. The most studied anatomical variations are those of the sciatic nerve, especially in relation to the piriformis muscle [30,31,32]. The most common variant of the sciatic nerve exits the pelvis through the greater sciatic foramen below the piriformis muscle. It descends between the greater trochanter and the ischiatic tuberosity [32], with an overall prevalence of approximately 90%, with high variability between individual studies. The common peroneal nerve is more susceptible to injury during total hip arthroplasty or other hip arthroscopic procedures, especially in patients with specific anatomical variants [31]. Another forensically relevant anatomical variation in the nerves of the lower limb is represented by the superficial course of the common peroneal nerve at the fibular neck (1.2–3.4 mm from the surface), which explains the very high prevalence of traction injuries at this level, especially during knee dislocations, fractures of the proximal fibula, or fractures of the tibial plateau [33].

The median nerve in the upper limb is known to have significant interindividual variation in its origin and course. An abnormal formation of the median nerve, from more than two roots, is seen in 11.5–52% of all cases, depending on the study [34,35,36,37], and is much more common in males than in females (81.8% vs. 18.2%, respectively) [36]. In a landmark study on the anatomical variations in the median nerve in carpal tunnel syndrome, Lanz found 29 variations in 246 hands, which he classified as type I, variations in the course of the thenar branch; type II, accessory branches at the distal portion of the carpal tunnel; type III, high divisions of the nerve; and type IV, accessory branches to the carpal canal [38]. Poisel classified the thenar branches as extra-, sub- (and trans-ligamentous in 46%), sub-ligamentous (31%), and 21% of cases, respectively [39]. All these variants have a significant impact on surgical outcomes and diagnostic accuracy. Clinicians should remain aware of these variants, as some require careful and specific preoperative planning and intraoperative awareness to optimize patient outcomes [36].

Anatomical variations in peripheral nerves have significant implications for surgical planning and execution, particularly in nerve reconstruction. Understanding intraneural topography is paramount while performing fascicular reconstruction, as the arrangement of motor and sensory fascicules varies between individuals and along the course of individual nerves [3,40,41]. The feasibility of distal nerve transfer is highly dependent on understanding both normal anatomy and anatomical variants [19]. Surgeons should consider the topographic organization of the target nerves when planning transfer procedures, as success is highly dependent on the appropriate matching of donor and recipient nerve fascicles [19]. Neurolysis also requires a detailed understanding of the anatomical variants and microanatomy of the nerve to achieve optimal outcomes [42,43]. Failure to account for anatomical variants during neurolysis could lead to incomplete symptom relief, inadvertent injury to unaffected neural components, and increased litigation risk.

Regional anesthesia and diagnostic procedures are another area in which peripheral nerve variations have significant consequences. The overall arrangement of the nerve fibers affects the overall effect of nerve blockade and the risk of nerve injury during the procedure [44]. To decrease this risk, various methods may be used, such as ultrasound, which may properly identify the nerve location, evaluate the relative position of blood vessels, nerves, and muscles around the puncture site, ultrasound aided by AI with medical image fusion, and neurostimulation [45].

The physiological processes involved when damaged neural structures lead to clinical symptoms are sometimes directly influenced or mimicked by those caused by anatomical variants [46]. Table 1 and Table 2 provide examples.

## 3. Diagnostic Challenges in Traumatic PNI

The diagnostic algorithm for traumatic PNI is complicated by the complex anatomy of the peripheral nerves, highly variable injury patterns associated with frequent anatomical variations, and significant limitations of mainstream assessment methods. Recent advances in microsurgical techniques, imaging technologies, electrophysiologic studies, and overall understanding of nerve microanatomy have increased diagnostic accuracy, but significant challenges still occur, especially in non-reference medical centers. The complexity of the evaluation in traumatic PNIs resides in the need to assess multiple components, such as motor function, sensory integrity, pain patterns, structural damage, while also taking into account the timing of injury, the age of the patient, associated complications, the type of medical intervention that was performed etc. [44,78].

For a proper forensic analysis, it is critical to classify the severity of the injury, a task that is difficult in traumatic PNIs. Traditional classification systems, such as those developed by Seddon and Sunderland (Table 3), provide an essential framework for understanding the nerve injuries the patient suffers, but often fail to capture the intricacies of complex trauma cases. Sedon defines three grades of PNI: neurapraxia, axonotmesis, and neurotmesis. Neurapraxia is caused by relatively minor injuries (usually compression in traumatic PNIs, causing neurotubules’ and neurofilaments’ fragmentation and axonal edema) [79], leading to a temporary loss of motor and sensory function, usually in up to eight weeks. Axonotmesis is caused by moderate trauma (a more severe compression injury, a stretch injury, or direct nerve contusion), causing damage to both the axon and the myelin sheath. It leads to motor and sensory dysfunction distally from the point of injury. It may be objectively assessed using imaging techniques, electromyography (which shows a denervation pattern, with fibrillation potential and positive sharp waves from 10 to 14 days after the traumatic event), or nerve conduction velocity studies (revealing loss of nerve conduction distally, from three to four days after the traumatic event). Neurotmesis is the most severe form of traumatic PNI, leading to an injury of both the nerve and the encasing fibrous tissue. It is usually caused by nerve lacerations (knives, shards of glass, saw blades) or severe stretch (as in severe dislocations) and, in the military, by explosions [80]. It has the worst prognosis, with a decreased chance of (even partial) recovery.

A proper distinction between different grades often requires a detailed understanding of the microanatomy and highly specialized and trained radiologists or other specialized techniques.

Compression injuries typically pertain to Grade I nerve injuries or neurapraxia and are common in the carpal tunnel or cubital tunnel, owing to nerves passing through confined anatomical openings. Crossing between rigid bony structures leads to compression of blood vessels, with subsequent ischemia and myelin displacement at the injury site. Both motor and sensory functions may be lost due to acute or chronic excessive pressure, even though the anatomy of the nerve is still intact and axonal damage is absent. Recovery is complete, and function is resumed relatively quickly (weeks to years) [85].

Axonotmesis generally occurs in injuries where the elastic nature of the nerve and its stretchability are defeated by a stronger traction force, commonly known as traction injuries [86]. This happens because of the nerve’s undulating characteristics loss when traction is applied for a long time or with extreme force. At this point, the perineurium takes on pressure with its tensile properties. If the traction force persists with the same intensity, the perineurium eventually tears [87].

Transection injuries are usually the cause of neurotmesis or grade V injuries and occur when the nerve is interrupted following severe trauma at that level. This is generally caused by the action of a sharp object (such as a knife) and can also be observed in patients with shrapnel injuries. In this case, the anatomical nerve structure is severed and requires surgical treatment to regain full functionality [81,88].

Clinical examination remains a cornerstone in the proper analysis of traumatic PNIs, despite its inherent limitations. The classical clinical approach consists of manual muscle testing, sensory collapse testing, and pain evaluation [89]. Details are provided in Table 4. Manual muscle testing has difficulties distinguishing between different causes of weakness or detecting more subtle motor deficits. It may also be compromised by a lack of cooperation, especially in acute trauma settings [89]. The challenges are even more complex in mixed nerve injuries, with motor and sensory components affected differently. Sensory testing has difficulty separating different types of sensory loss and correlating clinical findings with specific nerve territories (more pronounced in some specific anatomical variations) [83,90,91]. The Symptoms Categorization—History Taking-Examination—Diagnostic Evaluations Approach has been proposed to overcome some of these limitations as a more structured approach to manage these diagnostic challenges; however, proper implementation requires significant training and expertise to be effective [92].

MR neurography (MRN) has emerged as a highly sensitive method for assessing traumatic PNIs in recent years [84]. It can not only correctly classify injuries but also correlate with functional outcomes. The diagnostic accuracy of MRN varies depending on the severity of the nerve injury and anatomical location. For example, in detecting root nerve avulsion in brachial plexus injuries, MRN has a high specificity (89%) but only a moderate sensitivity (68%) [94]. MRN is especially useful in differentiating Sunderland grade III from grade IV injuries through its ability to visualize fascicular architecture and perineurial integrity properly [84,95]. The sensitivity is especially high in detecting Sunderland IV-V injuries (reaching 83.3% for detecting inferior alveolar and lingual nerve injuries), and much lower for Sunderland I-III injuries (19.1%, for the same nerves) [96]. The high soft tissue contrast helps identify neuroma-in-continuity formation in neurotmetic injuries, a critical differentiator from lower-grade axonal injuries. This addressed a key limitation of electrophysiological studies, which often struggle to differentiate between intact but non-conducting fascicles and complete anatomical disruptions [97]. More advanced MRN protocols incorporate quantitative measures, including T2 hyperintensity, nerve cross-sectional areas, and diffusion tensor imaging parameters. These have shown significant promise in bridging the gap between structural assessment and functional prognosis. For example, persistent elevation of T2 hyperintensity beyond 6 months after trauma is associated with poor sensory recovery and the development of chronic neuropathic pain [98,99]. Diffusion tensor imaging-derived fractional anisotropy values below 0.3 at the injury site predict incomplete motor recovery [100,101]. The development of specialized sequences, such as 3D TSE STIR black-blood imaging, was shown to increase the visualization of PNI [96,102].

The main limitations of MRN are its high cost and limited availability, especially in resource-limited institutions [103]. A lower and variable sensitivity is also a highly relevant issue, especially in a medical–legal context where a near 100% diagnostic accuracy is needed. It has a poor reliability in detecting pseudomeningoceles as markers of nerve root avulsion, limiting its usefulness in specific types of PNIs [9,94]. It also has temporal limitations, as signal changes may persist after the initial traumatic injury, and it may not appear immediately after trauma [96].

High-resolution ultrasonography is increasingly used to evaluate traumatic PNIs, offering distinct advantages, such as real-time imaging, cost-effectiveness, and widespread availability [11]. It is beneficial for detecting injuries in superficial nerves (such as the superficial palmar nerves) [102]. Its novelty resides in its potential to visualize individual nerve fascicles and detect subtle architectural changes, which were previously only visible using histological examination [102,104]. It may also be used during reconstructive nerve surgeries, enabling direct contact imaging for enhanced structural resolution and real-time guidance during surgical procedures [105]. Numerous scientific articles have shown that ultrasound (US) can correctly identify patients with unfavorable surgical outcomes resulting from anatomical predisposition [54,106,107,108].

It may determine whether the nerves are compromised, tethered, or hypermobile in relation to neighboring structures. It allows for the easier identification of multi-segmental injuries because the entire longitudinal course of the nerve can be evaluated in a single examination. It may also detect neuropathic pain, locate the nerves causing the symptoms, and identify the exact anatomical area responsible for a specific symptom [11]. Despite its obvious advantages, this procedure has significant limitations. The procedure has decreased sensitivity and specificity in deeper-located nerves or patients with poorer acoustic windows due to obesity or edema [109]. It also has a reduced sensitivity for detecting mild nerve injuries, the diagnostic accuracy being highly dependent on the degree of neural damage [110]. Operator dependency is also a significant limitation, as the sensitivity and specificity vary according to the experience or skill of the personnel [110]. There is an incomplete correlation between ultrasound findings and functional outcomes, especially when structural abnormalities do not correspond to clinical disabilities. Also, sometimes there is a poor correlation between ultrasound abnormalities and electrodiagnostic studies [102].

Nerve biopsy may sometimes be useful as a diagnostic tool in forensic pathology, but its use is currently limited due to its increased invasiveness compared to other methods. Current indications include suspected inflammatory neuropathies (for a differential diagnosis with traumatic PNIs), cases involving toxic neuropathies with unknown etiology, or when a hereditary neuropathy must be confirmed or excluded, and genetic testing is unavailable or inconclusive [111,112]. The timing of nerve biopsy is essential for maximizing the diagnostic yield. Optimal timing is between two and six weeks after the injury, allowing enough time to develop characteristic histological changes while avoiding secondary changes that potentially obscure the primary pathology [111]. Post-mortem, nerve biopsies should be performed as early as possible to minimize post-mortem artifacts caused by autolysis or putrefaction [113].

Electrodiagnostic studies (such as nerve conduction studies or electromyography) have long been considered helpful for properly assessing PNI.

All patients with PNI must be evaluated using motor conduction studies (MCS), sensory conduction studies (SCS), and electromyography (EMG) of the involved muscle. The parameters measured during MCS and SCS are distal latency, amplitude, and velocity [114].

Although they are included in the standard protocol for evaluating PNIs, electroconduction studies have low sensitivity and specificity (30–65%) [115,116]. In addition, their value is time-constrained, as immediately after the injury, they may not reveal the full extent of nerve damage, potentially causing false-negative results in the acute phase [78]. This may lead to a delayed diagnosis, causing a miss of the optimal window for surgical intervention in complete nerve disruptions. They also have difficulties in properly evaluating mixed nerve injuries, providing a comprehensive assessment of muscular function, and precisely identifying the anatomical location of the injury [89].

## 4. Prognosis

The prognosis depends on the patient’s age, mechanism of injury, and associated vascular and soft tissue injuries [117]. Although peripheral nerve injuries are not life-threatening, they may lead to a considerable decline in the patient’s quality of life [118]. The main elements that altered the prognosis of the patients are presented in Table 5.

## 5. Medical Legal Framework for the Evaluation of PNIs

The medicolegal evaluation of PNIs should begin with a proper identification and characterization of the cause (either trauma or an iatrogenic injury), the prejudice, which in turn should be quantified appropriately, and end with a proper evaluation of the causation link between them [129]. Adequate establishment for causation in PNIs requires a deep understanding of multiple factors, including mechanisms of injury, anatomical vulnerability, anatomical variations, physiopathology of injury and recovery, and temporal relationships between the event of interest and the effect. PNIs may be caused by various mechanisms, including compression, penetrating trauma, stretch, and ischemia, each associated with distinct patterns of damage and recovery [10,130]. These mechanisms should be correlated with documented clinical, imaging, and electrophysiological findings. This task is exponentially more complex in cases involving preexisting conditions or multiple potential causes of a particular nerve dysfunction. A more structured approach to evaluation is preferable whenever available, such as the SHED approach [131]. An overview of the general framework for PNI evaluation in a forensic context is presented in Table 6.

## 6. Causation and Timeline Determination in Traumatic PNIs

To establish a causal link in PNIs, experts should have a comprehensive understanding of the mechanisms of injury and their relationship with different patterns of nerve damage. The expert should correlate the proposed mechanisms with the observer pattern of nerve alteration and determine whether the relationship is scientifically plausible.

Iatrogenic nerve injuries are especially challenging to determine because they occur during otherwise appropriate medical procedures, and their overall frequency is not low. For example, femoral nerve injuries have an iatrogenic etiology in up to 60% of all cases, occurring during different types of surgical interventions (abdominal, pelvic, orthopedic, urologic) [133]. They may be caused by mechanical damage due to stretching, compression due to incorrect surgical positioning, thermal injury due to the use of electrocautery, or chemical injury caused by local anesthesia. Each mechanism leads to a distinct pattern of nerve dysfunction, which can be identified using clinical, electrodiagnostic, and imaging evaluations. An example of an iatrogenic femoral injury is presented in Table 7. Postmortem, the temporal sequence of changes occurring in nerves may be established using histological, electron microscopy, and molecular biology techniques. Electron microscopy is especially useful for detecting early changes that can be used for timeline determination. A study by Pieri et al. found that, at 12 h after death, a relevant loss of osmium affinity, with persistent myelin sheath and internodal tract, and visible, symmetrical, Schmidt–Lanterman clefts. On transversal sections, there is an initial loss of the myelin structure and the formation of small vacuoles. At 24 h, the osmium loss in the paranodal tract was apparent, myelin being flocculent and granular at this level, and the Schmidt–Lanterman clefts still symmetrical, but broader and longer. On transverse sections, up to 36 h after death, small elliptical splits were present within the myelin sheath, and small, osmiophilic granules were present near these discontinuities. Between 37 and 48 h, they are identifiable, aggregating in the axon, and bulging splits, especially in larger fibers. At 48 h, the osmium loss at the paranodal tract was more significant, and the Schmidt–Lanterman clefts were significantly lengthened. On transverse sections, after 48 h, a complete collapse of the internal myelin layers, which are fragmented in lamellar configurations, is apparent [134]. Vacchiano et al. used postmortem cholesterol levels from the median nerve to evaluate the postmortem interval from 20 to 136 h postmortem [135], with initially promising results, but the technique is more challenging to fully implement in forensic practice. Some of these alterations also occur after trauma. To properly differentiate them, we should evaluate the pattern of damage—traumatic injuries show a more organized pattern, with specific histological hallmarks, while postmortem changes show a diffuse, unorganized, non-specific degradation. Traumatic injuries show characteristic hallmarks such as the presence of an organized inflammatory response, specific patterns of axonal injuries such as retraction balls, and localized hemorrhage, which are clearly distinct from autolytic changes [136]. The presence of organized scar tissue formation, or specific patterns of Wallerian degeneration, is also typical for antemortem injuries [134,137].

The temporal relationship between traumatic/iatrogenic events and symptom onset provides highly relevant information for establishing causation. Although PNIs lead to immediate symptoms, the full extent of neurological dysfunction may not be clear until days or weeks later. Understanding the usual timeframe is necessary to differentiate these injuries from chronic degenerative conditions or other causes of nerve dysfunction. Preexisting conditions should also be carefully considered, as they may predispose patients to PNIs or mimic them. Anatomical variations should be considered, as they could make some individuals more prone to iatrogenic damage [139].

Regeneration timelines and recovery patterns are also essential for proper forensic analysis. Peripheral nerve regeneration is a slow process, typically requiring more than three months for axons to regenerate to distal target organs. During this time, the muscles undergo atrophy [140,141]. The usual regeneration rate is 1–2 mm daily, which helps assess recovery timelines based on the distance from the injury site to the target organ (s). In this regard, the classifications of Seddon and Sunderland can be used, even though they have some limitations, as mentioned above. Also, one must consider that recovery patterns differ based on the patient’s age, injury mechanism, anatomical location, and time to treatment [142]. Electrodiagnostic studies may provide objective measures of nerve regeneration, which may help track recovery times and predict functional outcomes. They may prove a progressive improvement, a plateau, or even a worsening case in which the causation nexus could be more complex, potentially involving medical malpractice [136,137,138,139].

Electromyographic temporal patterns may be used to assess the timeline. After PNI, Wallerian degeneration is initiated distal to the injury site, triggering electrophysiological changes detectable via EMG (Table 8).

MRN may also be extremely useful in establishing a timeline for PNIs. Following axonal injury, the blood–nerve barrier breaks down, triggering endoneurial edema, which is detectable through increased T2 signal intensity within 6–48 h, preceding Wallerian degeneration, which is characterized by progressive distal nerve hyperintensity and volume loss. Details are presented in Table 9.

## 7. Malingering in Traumatic PNIs

The American Psychiatric Association defines malingering as the intentional production of false or grossly exaggerated physical or physiological problems, usually externally motivated (avoiding military duty, work, obtaining financial compensation, avoiding/evading criminal prosecution, or obtaining controlled substances [147]. From both clinical and medico-legal perspectives, individuals who malinger often present symptomatology that is incongruent with the documented organic substrate, as confirmed by multidisciplinary evaluation, including neuropathic pain, unexplained muscular weakness, non-anatomical sensory deficits, allodynia, and hyperalgesia [148].

Malingering remains a challenging issue in medico-legal practice, mainly because there are sometimes significant difficulties in separating it from genuine psychological suffering. One of the most ethically and legally sensitive aspects of the medicolegal assessment is the potential misclassification of a genuine patient as a malingerer, which can seriously compromise both the quality of medical care and the integrity of legal proceedings [149].

Research has shown that current psychometric tools for detecting malingering have limited sensitivity and specificity, especially in psychiatric populations [150]. Therefore, a contextualized case-by-case analysis is essential, one that integrates the clinician’s experience with the available objective assessment tools to ensure accurate and ethically sound evaluations [151,152].

The differential diagnosis of posttraumatic neurological deficits often entails a multifaceted approach using both objective clinical methods and subjective evaluations. Quantifying neurological deficits is a critical aspect of forensic evaluations when assessing the severity of peripheral nerve injuries resulting from trauma [153].

This procedure mainly utilizes objective assessment methods, especially within medico-legal frameworks that depend exclusively on the objective evaluation of traumatic injuries and their effects, to create a definitive link between neurological harm and its influence on the claimant’s functional abilities [154].

Objective methods, such as electrodiagnostic tests and advanced neuroimaging, including high-resolution ultrasound and magnetic resonance neurography, provide quantifiable data regarding affected nerves’ anatomical and functional integrity, thus facilitating diagnosis, severity assessment, and prognosis [155,156,157]. Simultaneously, subjective methods, such as patient-reported outcome measures and standardized pain assessment tools, are essential for capturing nerve injury’s functional and psychosocial impact from the patient’s perspective, especially in chronic or neuropathic pain contexts [158,159,160].

A few approaches have been used to assess neuropathic pain, such as verbal rating scales, numeric rating scales, and visual analog scales, which are easy to use in clinical practice. More complex questionnaires are available that measure pain intensity and quality [161].

However, these questionnaires are usually complicated to include in practice because they are time-consuming and burdensome to analyze. The most frequently used and cited pain questionnaires are the McGill Pain Questionnaire, Self report-Leeds Assessment of Neuropathic Symptoms and Signs; RAND-36; Disability of Shoulder, Arm and Hand, designed to assess three components of pain (i.e., sensory, affective, and evaluative), but with little study evidence to support the benefit of their use [162,163].

Accurate techniques for assessing somatosensory function are essential in legal medicine scenarios concerning neuropathic pain, nerve injury from trauma, or complex regional pain syndrome. Quantitative Sensory Testing, along with electrophysiological assessments, are frequently employed methods that offer insights into the state of the sensory system [164]. Quantitative Sensory Testing is a psychophysical method used to assess sensory thresholds by examining reactions to controlled stimuli related to mechanical, thermal, and pain sensations, providing insights into the function of small-diameter myelinated (Aδ) and unmyelinated (C) fibers, which helps in the early detection of sensory deficits [165]. Quantitative Sensory Testing comprises static assessments that measure thresholds, identify hyperalgesia or hypoesthesia, and dynamic evaluations that indicate the central mechanisms of pain modulation [166,167]. Nonetheless, this method is subjective, reliant on patient cooperation and cognitive status, and does not permit precise localization of lesions [168].

## 8. Conclusions

The medical legal evaluation of PNIs needs a structured, interdisciplinary approach, integrating anatomical clinical, imaging and medical-legal principles and theories. This review aims to consolidate the foundational knowledge needed for an accurate diagnosis, the grading of the severity and the assessment of the timeline of the PNIs, which are often complex, and often further complicated by anatomical variations and limitations of diagnostic tools.

The tabulated data and tabulated framework provided here may be used as practical tools for medical-legal experts to increase consistency, reproducibility and objectivity in complex, expert analyses. Are emphasized the importance of recognizing anatomical variants, using appropriate imaging and electrodiagnostic methods, and understanding nerve regeneration patters associated with timeline markers, which are crucial for causation analysis and a proper differential diagnosis.

By promoting a systematic, scientifically grounded methodology, this review aims to further improve the activity of medical-legal experts by increasing the accuracy and defensibility of their opinions, therefore contributing to a more equitable, and evidence based judicial outcome.

## Figures and Tables

**Table 1:** Anatomical variations leading to lower limb nerve compression (Based on [46,47,48,49,50,51,52,53,54,55,56,57]). Are detailed the nerve, common compression sites, and anatomical variations leading to compression.

Nerve	Common Compression Sites	Anatomical Variations Causing Compression
Sciatic Nerve	- Piriformis muscle - Greater sciatic foramen - Ischial tuberosity region	- Nerve passes through the piriformis muscle (15–20% of cases) - Bifid piriformis with nerve passage between muscle bellies - High sciatic nerve division with separate exits - Fibrous bands across the sciatic notch - Anomalous fibrous bands across the sciatic notch
Common Fibular Nerve	- Fibular head and neck - Fibularis longus arcade - Lateral compartment fascia	- A more medial course around the fibular head - A thick fibrous arcade of the fibularis longus - Accessory muscle of the fibularis longus - Anomalous fibrous bands in the lateral compartment
Superficial Fibular Nerve	- Lateral compartment exit point - Deep fascia perforation site - Ankle region	- A high fascial exit point (more proximal) - Multiple perforations through the fascia - A thick lateral compartment fascia - Aberrant muscle insertions resulting in fascial bands
Deep Fibular Nerve	- Anterior compartment - Extensor retinaculum - First web space	- Accessory muscle of the extensor digitorum longus - Anomalous course of the extensor hallucis longus - A thick superior extensor retinaculum - Variant branching patterns noted at the ankle
Posterior Tibial Nerve	- Tarsal tunnel - Flexor retinaculum - Medial calcaneal branches	- Accessory muscle of the flexor digitorum longus - Anomalous insertion of the plantaris - A thickened flexor retinaculum - Aberrant vessels related to the posterior tibialis - Variant branching of the medial plantar nerve
Lateral Femoral Cutaneous Nerve	- Anterior superior iliac spine - Inguinal ligament - Sartorius muscle origin	- Nerve passes through the inguinal ligament - Multiple divisions of nerves - Aberrant course through the sartorius - Anomalous fascial attachments to the anterior superior iliac spine
Obturator Nerve	- Obturator foramen - Adductor canal - Thigh adductors	- An accessory obturator nerve - Variant anatomy of the obturator foramen - Anomalous insertion of the adductor muscle - Fibrous bands noted within the obturator canal
Femoral Nerve	- Inguinal ligament - Iliacus muscle - Adductor canal	- Variant attachments of the inguinal ligament - An accessory slip of the iliacus muscle - Anomalous positioning of the femoral vessels - A thickened iliopectineal fascia
Sural Nerve	- Lateral border of foot - Fifth metatarsal base - Achilles tendon region	- A variant formation (medial vs. lateral sural) - An anomalous branching pattern - Aberrant fascial attachments - A variant relationship to the short saphenous vein
Saphenous Nerve	- Adductor canal - Medial knee - Medial malleolus	- A high branching pattern of the infrapatellar branch - Variant course through the sartorius - Anomalous fascial planes in the adductor canal - Aberrant relation to the saphenous vein

**Table 2:** Anatomical variations leading to upper limb nerve compression (based on [37,46,58,59,60,61,62,63,64,65,66,67,68,69,70,71,72,73,74,75,76,77]. Are detailed the nerve, common compression sites, and anatomical variations leading to compression.

Nerve	Common Compression Sites	Anatomical Variations Causing Compression
Radial Nerve	- Humeral shaft (esp. distal third) - Lateral epicondyle - Axilla	- Lateral intermuscular septum: separates the RN from the humerus by 1–5 cm - Three-headed extensor carpi radialis longus: medial head fused with extensor carpi radialis brevis - Four-bellied biceps brachii: compresses the RN near the greater tubercle - Accessory belly of pronator teres: lateral/distal compression - Axillary arch: compression in the axilla
Median Nerve	- Proximal forearm (pronator teres) - Carpal tunnel	- Gantzer muscle: compresses the anterior interosseous nerve (the Kiloh-Nevin syndrome) - Innervation to coracobrachialis (rare, cadaveric) - Anterior interosseous origin under the arch of the pronator/flexor - Accessory brachial muscle: crosses the MN at the elbow - Palmaris profundus exiting through the carpal tunnel - Accessory belly of the first lumbrical: into the carpal tunnel - Pronator teres with two heads, with compression at the distal insertion - Struthers’ ligament: fibrous band at the elbow - Axillary arch: compression in the axilla - Supracondylar process: congenital bony prominence
Ulnar Nerve	- Cubital tunnel (elbow) - Guyón canal (wrist)	- Extensor digitorum brevis manus: compression near the 5th metacarpal bone - Accessory abductor digiti minimi: Guyón canal syndrome - Variant flexor digiti minimi brevis fascicle: compression near the 5th MCP - Anconeus epitrochlearis: compression in the epitrochlear region (common)
Suprascapular nerve	- Suprascapular notch, - Spinoglenoid notch	- Narrow or V-shaped suprascapular notch - Ossified or thickened superior transverse scapular ligament - Ganglion cysts at the spinoglenoid notch - Variant course of nerve around scapular spine
Axillary nerve	- Quadrangular space (shoulder)	- Narrow quadrangular space - Fibrous bands within the space - Ganglion cysts or hematomas - Hypertrophic teres minor or major muscles
Musculocutaneous nerve	- Coracobrachialis muscle (arm)	- Additional or aberrant muscle heads (e.g., accessory coracobrachialis, four-headed biceps) - Communication with the median nerve (rare) - Fibrous bands within or around the muscle

**Table 3:** Seddon and Sunderland classifications for nerve injuries (based on [81,82,83,84], their main symptoms and MRN characteristics.

Classification	Grading	Details	Symptoms (for Seddon) and MRN Characteristics (for Sunderland)
Sedon	Neurapraxia 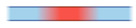	- the mildest form, with minimal structural damage - complete recovery in a relatively short period of time - caused by a focal demyelination at the site of injury, without axonal disruption - etiology > compression, causing prolonged ischemia from excess pressure, or nerve stretching, without Wallerian degeneration	- pain - muscle weakness - numbness - altered proprioception
	Axonotmesis 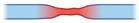	- damage to both the axon and the myelin sheath, without structural alterations of the endoneurium, perineurium, or epineurium - may be associated with Wallerian degeneration - complete recovery, in a longer timeframe	- pain - muscle wasting - complete function loss (motor, sensory, sympathetic)
	Neurotmesis 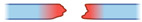	- the most severe form - leads to the disruption of both axons and encasing connective structures	- complete anesthesia - muscle wasting - complete function loss (motor, sensory, sympathetic)
Sunderland	I (neurapraxia)	- focal demyelination without axonal damage	Increased T2 signal intensity for nerves, normal intensity for muscles
	II (axonotmesis)	- axonal damage with intact endonerium	Increased T2 signal intensity, diffusely enlarged.
	III (axonotmesis)	- axonal and endoneurial damage, with intact perineurium - recovery can be slow and dependent on the degree of scarring and fascicular involvement	Fascicles enlarged or effaced (edema). Muscles—denervation
	IV (axonotmesis)	- axonal, endoneurial, and perineurial damage, with intact epineurium - spontaneous recovery does not occur, and surgical intervention with grafting is needed	Nerves—focally enlarged, with heterogenous signal intensity, inconstant underlying diffuse abnormality, fascicles disrupted with heterogenous signal intensity. Muscles—denervation
	V (neurotmesis)	- complete nerve transection - poor prognosis, surgical intervention is needed	Complete nerve discontinuity, inconsistent hemorrhage, inconsistent fibrosis in the nerve gap, and end-bulb neuroma proximally. Epineurial thickening. Muscles—denervation

**Table 4:** Clinical examination in PNIs (based on [89,93]).

Test	Details
Manual muscle testing	- aims to identify specific weakness patterns, helping in the proper localization of the nerve injury - muscle strength is graded from M0 (no muscle power) to M5 (full muscle power) - evaluation is performed using grade M4 (power against moderate resistance)
Sensory collapse test	- the patient must resist applied force before and after the examiner scratches/touches the skin over the suspected nerve compression site - a focal nerve compression causes a temporary loss of muscle strength
Pain testing	- allodynia is evaluated at the compression site

**Table 5:** Prognostic factors in PNIs, useful for a proper evaluation of the medical-legal consequences of these injuries.

Prognostic Factor	Details
Grading	- Neurapraxia has a favorable prognosis, with most patients recovering within two to three months [119]; - Axonotmesis presents a variable prognosis, largely dependent on the ability of axons for regeneration [120]; - Neurotmesis offers minimal chances for spontaneous recovery without surgical intervention [121].
Injured nerve	- For injuries of similar severity, the surgical outcome is best for radial nerve lesions, followed by median, and then ulnar [34].
Age	- For sensory recovery, younger patients have better outcomes compared to older patients [35,36].
Mechanism of injury	- Sharp transections have better surgical outcomes compared to crush or traction injuries; - Chronic compression may lead to progressive, irreversible axonal degeneration [121,122].
Defect length	- Short-segment nerve grafts have superior functional outcomes compared to long-segment grafts [123].
Injury location	- The surgical outcome is poorer if the injury is closer to the proximal end [124].
Time to repair	- Earlier surgical repair is correlated with an improved prognosis. A delay of more than six to twelve months significantly reduces the likelihood of a meaningful recovery [125]; - A delay of just six days may cause 1% loss of function [126], as nerve regeneration progresses at an estimated rate of 1–2 mm/day.
Repair method and materials	- Autologous nerve grafts are the gold standard, and generally yield the most favorable outcomes; - Alternate repair materials, such as non-degradable silicone tubes, polytetrafluoroethylene (PTFE) or polyethylene conduits, as well as biodegradable constructs such as vein, skeletal muscle, and acellular nerve allografts [127,128] tend to have inferior clinical results.

**Table 6:** Medical–legal framework for PNI evaluation (based on [8,129,132]).

Aspect	Details
Documentation Requirements	- Clinical records should be contemporaneous, accurate, and detailed, to support medical–legal analysis - They should include: neurological examination, photographs of wounds and surgeries, functional assessment, and, if available, modern imaging techniques (MR neurography, high-resolution ultrasonography)
Legal Admissibility of Evidence	- Evidence should meet legal criteria: reliability, relevance, adherence to scientific standards - All imaging techniques and electrodiagnostic tests must be evaluated by highly trained specialists, and should follow standardized, available protocols or guidelines
Limits of current evaluation techniques	- Nerve conduction studies performed too early could miss the extent of the injury - Clinical evaluation has a limited sensitivity and specificity - MRN is expensive and not highly available; the expert should not require it as mandatory if the costs are too burdensome for the subject, and if there are alternate methods able to provide a relatively similar degree of certainty
Clinical Guidelines vs. Legal Standards	- Clinical guidelines and standards for diagnosis and treatment have useful recommendations, but they do not define legal standards of care; therefore, they may be, in particular circumstances, circumvented - If the physicians do not fully respect the guidelines, the expert should evaluate why, and also if this potential breach has actually had an effect on the evolution of the disease or the standard of proof
Deviations from the standard	- Potential deviations from the standard of care should be evaluated to determine whether they can be classified as negligence, taking into account the context of the patient, trauma, the limits of the resources available, and the evolving standards - The evaluations should be made in relation to the standards at the moment of injury/treatment, not at the moment of the evaluation, as they might change

**Table 7:** Patterns of femoral nerve dysfunction based on the mechanisms of injury (based on information from [133]. MRC—Medical Research Council muscle grading system [138], NCS—nerve conduction studies, EMG—electromyography.

Type of Damage	Mechanism	Motor Function	Sensory Function	Reflexes	Pain	NCS	EMG	Timeline	Prognosis
Neuroapraxia	Compression, Stretching, Traction	Partial quadriceps weakness (MRC 2–4), preserved hip flexion	Mild-moderate hypoesthesia, tingling	Reduced patellar reflex	Minimal, discomfort	Normal distal to the lesion	Minimal denervation, preserved recruitment	Changes in 7–10 days	Excellent—recovery in weeks to 3 months
Axonotmesis	Partial Nerve Injury with Axonal Damage	Moderate-severe quadriceps weakness (MRC 1–3)	Marked hypoesthesia to anesthesia in L2–L3	Severely diminished or absent	Moderate neuropathic pain	Absent responses after 9+ days	Fibrillation, denervation in 2–4 weeks	Differentiated from neuropraxia after 1 month	Good—recovery in 6–12 months
Neurotmesis	Complete Nerve Transection	Complete paralysis (MRC 0)	Complete anesthesia in the femoral distribution	Absent	Severe, chronic, possible	Absent proximal and distal	Severe denervation, electrical silence	No improvement by 2–3 months	Poor—needs surgical repair
Ischemic	Vascular Compromise	Variable, can be mild to severe	Pronounced, burning, dysesthetic	Proportional to nerve damage	Burning worsens with position	Slowed velocities, patchy blocks	Patchy denervation, progressive	Longer recovery	Variable—depends on ischemia severity
Heat	Thermal Injury (e.g., electrocauter)	Severe weakness (MRC 0–2)	Profound loss, skin changes	Absent	Severe, permanent syndrome	Severely abnormal/absent	Extensive denervation, poor recovery	Limited recovery	Poor—permanent deficits
Toxic	Chemical Injury (e.g., ethanol)	Variable, selective involvement	Chemical burn-type loss	Reduced or absent	Severe, resistant to treatment	Sensory responses are more affected	Mixed pattern of denervation	Depends on the agent and duration	Variable—sensory, often incomplete

**Table 8:** Chronology of EMG changes in PNI (MUAP—Motor Unit Potential Evolution). Based on [143,144,145,146]. Are detailed the main EMG findings, and their clinical and forensic implications.

Timeline	EMG Findings	Clinical/Forensic Implications
0–3 Days	Loss of neural input; membrane instability begins	No EMG changes; baseline study possible
10–14 Days	Emergence of fibrillation potentials and positive sharp waves	Confirms axonal injury; supports timing of denervation
21–28 Days	Peak fibrillation density	Reflects maximal denervation; useful in injury dating
>6 Weeks	Persistent fibrillations	Suggests failed reinnervation or chronic axonal injury
Weeks 2–12	Polyphasic MUAPs; reduced recruitment	Indicates early reinnervation via collateral sprouting
>3 Months	High-amplitude, long-duration MUAPs; reduced fibrillation	Confirms reinnervation; evaluates recovery and standard of care

**Table 9:** Chronology of MRN signal changes in PNI (DWI—diffusion weighted imaging; Ktrans—volume transfer constant; DTI—diffusion tensor imaging; ADC—apparent diffusion coefficient; 3D PSIF—Reversed Fast Imaging with Steady-State Free Precession; CSA—cross-sectional area).

Phase/Timeline	MRN Findings	Pathophysiological Interpretation
0–24 h	Focal nerve swelling with T2 hyperintensity (↑ ≥ 30%)	Blood–nerve barrier disruption, early edema
24–72 h	Dynamic contrast-enhanced MRI shows ↑ Ktrans (↑ 40%)	Blood–nerve barrier leakage
3–7 days	DWI shows restricted diffusion (ADC ↓ 15–20%)	Early Wallerian degeneration
Week 1–2	Peak T2 hyperintensity (2.5× baseline)	Macrophage infiltration, edema peak
Week 3–4	DTI shows ↓ fractional anisotropy (↓ 50%), ↑radial diffusivity (↑ 200%)	Axon loss and demyelination
Week 5–6	3D PSIF shows neuroma with ‘coiling’ morphology	Neuroma formation and perineural fibrosis
>6 Weeks	T2 hypointense cores, ↓ nerve CSA (30–40%), fatty replacement	Chronic phase: atrophy, failed regeneration
>6 Weeks	DTI shows normalized axial diffusivity	Successful reinnervation and fascicular restoration

↓—decreased; ↑—increased.

## Data Availability

Not applicable.
